# *Alternaria alternata* Pathogen from *Cuscuta japonica* Could Serve as a Potential Bioherbicide

**DOI:** 10.3390/jof10070494

**Published:** 2024-07-17

**Authors:** Yinglong Liu, Ayesha Ahmed, Shahzad Munir, Lei Chen, Pengfei He, Yueqiu He, Ping Tang, Baohua Kong, Yixin Wu, Pengbo He

**Affiliations:** 1 State Key Laboratory for Conservation and Utilization of Bio-Resources in Yunnan, Yunnan Agricultural University, Kunming 650201, Chinaynfh2007@163.com (Y.H.);; 2 College of Agronomy and Life Science, Zhaotong University, Zhaotong 657000, China

**Keywords:** dodder, biological control, *Alternaria*, bioherbicide

## Abstract

Dodder (*Cuscuta* spp.) is a dangerous parasitic plant that causes serious damage to crop production and is challenging to eliminate. Herbicide application is a common strategy to control dodder in the field, but it is costly, ineffective, and further results in hazardous outcomes. Therefore, our study aims to identify the potential pathogens in naturally occurring dodder infections which may provide efficient biocontrol options. In this regard, the pathogens were isolated from the infected plants, their pathogenicity was validated through inoculation, and the optimal culture conditions for their growth were identified by determining the pathogenicity difference. The pathogenicity range was determined in vitro using the leaves of common horticultural plants and crops. Furthermore, a small range of horticultural plants parasitized by *Cuscuta reflexa* in the field were inoculated with the pathogen to determine their biosafety and biocontrol potential, and the pathogens were identified by morphological and molecular characterization. We found 7 strains that were isolated after pathogen enrichment culture. Among them, Cbp6 and Cbp7 showed the highest pathogenicity against *C. reflexa*. After testing the inoculation of more than 50 species of plants, only 9 species showed varying degrees of lesions on leaves, which proved the high biosafety for common plants. Field spraying of these pathogens showed a good control effect on *C. reflexa* after 21 days; the disease severityreached 66.0%, while its host plant did not display obvious symptoms. In conclusion, the pathogens Cbp6 and Cbp7 were identified as *Alternaria alternata*, and the results of this study provide a theoretical basis for the biological control of dodder.

## 1. Introduction

Dodder (*Cuscuta* spp.) is a typical holoparasitic plant belonging to the family Convolvulaceae, order Solanales [1,2]. Due to evolution, it is leafless and rootless, and although it contains trace amounts of chlorophyll, it no longer possesses photosynthetic abilities. Instead, it penetrates the host stem with a large number of haustoria, forming vascular connections to feed on the host’s nutrients and water. It establishes a bidirectional link to transport signaling substances, viruses, secondary metabolites, proteins, mRNAs, small RNAs, and lncRNAs in the host plant which severely limits its growth and development [3,4,5,6,7]. With a wide host range, they can parasitize a variety of herbaceous and woody plants, including common horticultural plants and important crops [8]. Dodder not only restricts plant growth but also acts as a source of various pathogens which is concern for global agriculture. *Cuscuta* spp. are reported to be infectious and can transmit over 50 types of viruses. On the other hand, it is also reported that species such as *C. campestris*, *C. australis*, and *C. japonica* can limit the growth of *Mikania micrantha*, *Humulus scandens*, and *Ambrosia trifida* within their native ranges. Thus, some of the dodder species exhibit a degree of biological control potential against other invasive weeds [7,9,10]. Furthermore, they are rich in flavonoids and other plant molecules which promote antiviral, antibacterial, and anti-inflammatory activities [11,12]. Nonetheless, they causes huge losses in agricultural production and has a negative effect on biodiversity that far outweighs its applied value.

Dodder uses invasive structural haustoria to connect with the host vascular tissue, thus forming a stable parasitic state; therefore, the damage is persistent, widespread, serious, and difficult to eradicate [13]. Currently, few host plants are completely resistant to dodder [14,15]. Herbicides and other chemical pesticides have shown good control effects against dodder but have an adverse effect on the growth and development of host plants and the ecological environment, and also lead to development of resistance [16,17]. In addition, although the manual removal is beneficial, it is time-consuming, laborious, and expensive, so there is currently no efficient control method. 

Biological control methods utilizing microbes that are naturally pathogenic to weeds is a safe, economical, and environmentally sustainable option [18]. For instance, *Alternaria destruens* L. Simmons strain 059 registered as Smoulder^®^, is a fungus that is pathogenic towards dodder species. It was isolated as an indigenous pathogen from swamp dodder and has been used to control several species in crops such as alfalfa, dry bog cranberry, carrot, pepper, tomato, eggplant, blueberry, and woody ornamentals [19]. However, such microbial resources against weeds, especially dodder, are scarce. Therefore, our study aimed to identify microbes that are pathogenic to dodder species to devise an environmentally friendly and cost-effective control strategy.

To find natural infection, a field visit to Huili County (27°84′38.57″ N, 102°33′94.57″ E), Sichuan Province, China, was conducted in March 2023, where *C. japonica* parasitizing *Ligustrum quihoui* was found to be dying of the disease. The symptoms, such as large areas of black spots on the stalks, and browning, dehydration, and atrophy on the upper end of the stem, were recorded. It was hypothesized that these symptoms might be caused by the plant pathogens. To isolate and explore the pathogens, samples were collected from the field and healthy plants were inoculated with the possible pathogens to confirm their pathogenicity. Diseased tissues were isolated, and pathogens were identified by Koch’s postulates. The pathogenic strains identified may provide effective means to develop bioherbicides against dodder species.

## 2. Materials and Methods

### 2.1. Diseased Sample Collection and Pathogen Enrichment Culture

Naturally infected *C. japonica* was collected from Huili County (27°84′38.57″ N, 102°33′94.57″ E), and healthy *C. reflexa* was collected from Kunming City, Yunnan Province (25°12′82.65″ N, 102°74′89.22″ E). In order to verify the pathogenicity and to enrich pathogens, infected *C. japonica* and healthy *C. reflexa* were mixed and placed in a plastic bag to maintain moisture. Healthy dodder was used as a control and kept in a 25 °C incubator for 7 days. During this period, the degree of disease incidence was observed. Afterward, the length of the black-brown wilt spot and the total length were measured, and the length of the lesions and the disease severity were calculated according to the following formula [20]:

Disease severity (%) = (total length of lesions/total length of investigated dodder stem) × 100

### 2.2. Pathogen Isolation and Pathogenicity Determination

Pathogens were isolated from naturally infected *C. japonica* collected from the field and diseased *C. reflexa* after enrichment culture. The infected tissues were rinsed under running water, soaked in 75% alcohol for 30 s for surface disinfection, and rinsed three times with sterile water to remove the alcohol. The infected stems were cut into small squares of 1–3 mm length and then transferred to potato dextrose agar (PDA: 200 g/L potato, 20 g/L glucose, 15 g/L agar) medium plates containing rifampicin (Solarbio, Beijing, China) and ampicillin (Solarbio, Beijing, China) at a concentration of 10 mg/L each, and then incubated at 28 °C for 1–3 days. The pure isolates were obtained according to the colony morphology. 

To determine the pathogenicity of the isolates (recovered from naturally infected *C. japonica* and inoculated *C. reflexa*), the stems of the healthy dodder (*C. reflexa*) were cut into 5 cm segments, and the surfaces were disinfected with 75% alcohol. Then, they were placed on the PDA plates containing the isolates with colony diameters of up to 2 cm, and incubated at 28 °C for 3 days. The appearance of black-brown lesions on the dodder stems and/or the growth of mycelium on the surface of the stems were used as the basis of pathogenicity, and the pathogenic isolates were screened out according to morbidity status. After the preliminary screening, the candidate strains were cultured on PDA plates for 7 days, and then the mycelium was gently scraped down and cultured for another 3–5 days to induce the production of a large number of conidia. The conidia were eluted with sterile water containing 0.2% (*v*/*v*) Tween-20, and the concentration was adjusted to 1 × 10^6^ spores/mL. Healthy *C. reflexa* with a total length of 5 m was placed in a plastic bag and uniformly sprayed with 10 mL of conidial suspension, while water was used as negative control; each treatment had 3 replicates. Afterwards, the plants were placed in a constant temperature incubator at 25 °C for 7–14 days. The lesions on the dodder stems were continually observed and photographed during this period to calculate disease severity. Following Koch’s postulates, the pathogen was re-isolated, and the pathogenicity was determined.

### 2.3. Determination of Differences in the Pathogenicity of Dodder Pathogens

Spore suspensions of highly pathogenic strains were prepared as described previously, and the spore concentrations were adjusted to 1 × 10^6^, 1 × 10^7^, and 1 × 10^8^ spores/mL. Healthy *C. reflexa* of 5 m in length was sprayed with 10 mL of spore suspension, or sterile water as a control; each treatment had 3 replicates. After 7 days of incubation, as described above, the length of the lesions was measured, the disease severity was calculated, and the pathogenicity of the different concentrations of spore suspensions was compared.

### 2.4. Physiological Characterization of Dodder Pathogens

To determine the optimal growth conditions and nutritional requirements of dodder pathogens, colony growth, and conidia production ability were examined using different media, pH values, and temperatures. Firstly, the pathogens were inoculated onto the center of PDA medium plates and incubated at 28 °C for 7 days. A 7 mm sterile perforator was used to perforate along the same circumference, and the mycelial blocks were inoculated onto the center of PDA, corn meal agar (CMA; Hopebiol, Qingdao, China), and malt extract agar (MEA; Solarbio, Beijing, China) medium plates (the diameter of the plate was 6.0 cm, and each plate contained 15 mL of medium). Each treatment had 3 replicates and each replicate consisted of 4 plates which were incubated at 28 °C for 3–5 days, after which the colony diameters were measured to compare the growth status of the pathogens. At the same time, the surface of the colonies on the plates cultured for 7 days were gently scraped and then cultured for another 3–5 days under dark conditions to stimulate the production of conidia. Each plate was washed with 15 mL of sterile water containing 0.2% (*v*/*v*) Tween-20 to wash off the conidia, and the number of conidia was then counted to determine the optimum medium for conidia production. To determine the optimum pH value for dodder pathogens, the pathogens were inoculated onto PDA medium with varying pH values of 4, 5, 6, 7, 8, 9, 10, and 11. Colony diameters and conidia production were measured after incubation under the same conditions as before. Additionally, the pathogens were inoculated onto PDA medium and incubated at different temperatures of 4, 16, 22, 28, 32, and 38 °C. Afterward, the colony diameters and conidia production were measured to determine the optimum growth temperature for dodder pathogens.

### 2.5. Determination of the Pathogenic Range of Dodder Pathogens

To evaluate the safety of dodder pathogens in common plants, the pathogenicity of the candidate pathogens on the leaves of common plants was determined by in vitro leaf inoculation. Host plant leaves of *Cuscuta* spp. were collected from Kunming city (25°12′82.65″ N, 102°74′89.22″ E), including *Hedera nepalensis* var. *sinensis*, *Rhododendron pulchrum*, *Osmanthus fragrans*, *Duranta erecta*, *Vinca major*, *Vinca major* cv. *variegata*, *Bougainvillea spectabilis*, and *Buxus sinica* var. *parvifolia*. Similarly, leaves from other common horticultural plants such as *Nandina domestica*, *Morus australis*, *Euryops pectinatus*, *Mucuna sempervirens*, *Fatsia japonica*, *Loropetalum chinense* var. *rubrum*, *Salvia japonica*, *Jasminum nudiflorum*, *Celtis sinensis*, *Eleutherococcus nodiflorus*, *Euonymus japonicas*, *Ternstroemia gymnanthera*, *Hypericum henryi*, *Cinnamomum camphora*, *Schefflera octophylla*, *Solanum pseudocapsicum*, *Salix babylonica*, *Rosmarinus officinalis*, *Clivia miniata*, *Iris ensata*, *Canna glauca*, *Ophiopogon bodinieri*, *Cerasus yedoensis*, *Phyllostachys sulphurea*, *Pharbitis nil*, and *Cycas revolute* were collected. Additionally, common fruit, vegetable, and other cash crops used in the study were *Citrus limon*, *Eriobotrya japonica*, *Morella rubra*, *Punica granatum*, *Diospyros lotus*, *Juglans mandshurica*, *Cucurbita moschata*, *Zea mays*, *Glycine max*, *Ipomoea batatas*, *Perilla frutescens*, *Raphanus sativus*, *Nicotiana tabacum*, and *Polygoni multiflori*. Healthy leaves of the above test plants were collected, while *C. reflexa* was used as a control plant. The spore suspension of the pathogen at a concentration of 1 × 10^8^ spores/mL was sprayed uniformly on both sides of the tested leaves, and sterile water was sprayed as a control; each treatment consisted of 3 replicates, and each replicate consisted of 3 leaves. After the treatments, all leaves were placed in clean plastic bags and incubated at 25 °C (light: darkness = 1:1; light intensity of 5000 lux) for 3–7 days. The pathogens were re-isolated from the infected leaves, which confirmed that the pathogenicity was verified by Koch’s postulates.

### 2.6. Determination of the Efficacy of the Pathogen against Dodder in the Field

After investigation, naturally infected *H. nepalensis* var. *sinensis* and *V. major* (6 plants each) parasitized by *C. reflexa* were located on the farm of Yunnan Agricultural University, Panlong District, Kunming City, Yunnan Province, China. To verify the control effect of the candidate pathogens on *C. reflexa* in the field environment and to determine biosafety in the host plants, a spore suspension of the pathogen at a concentration of 1 × 10^8^ spores/mL was uniformly sprayed onto the *C. reflexa* plants and the host plants in the field. Symptoms were observed at 7, 14, and 21 days after treatment; lesion length was measured and disease severity was calculated, while relative control effect was calculated according to the following formula [20]:

Relative control effect (%) = (disease severity in treatment group − disease severity in control group) × 100/(100 − disease severity in control group)(1)

### 2.7. Identification of Dodder Pathogens

Dodder pathogens were cultured on PDA plates, and characteristics such as appearance, morphology, and colony color were observed. Furthermore, structures such as mycelium, conidiophore, conidia, and chlamydospore were observed under the microscope. Meanwhile, the genomic DNA of the pathogen was extracted by the CTAB method and primer pairs ITS1/ITS4 [21], LR0F/LR5R [22], bRPB2-6F/bRPB2-7R [23], and EF1-1018F/EF1-1620R [22] were used to amplify the internal transcribed spacer (ITS) rDNA, large subunit ribosomal rRNA (*LSU*), RNA polymerase II second largest subunit (*RPB2*), and translation elongation factor 1α (*TEF-1α*) gene, respectively. The PCR reaction mixture contained 6.0 μL of buffer (2× Taq master mix, Novoprotein, Suzhou, China), 1 μL of forward and reverse primers, 11 μL ddH_2_O, and 1 μL genomic DNA. The amplification conditions were as follows: initial denaturation at 94 °C for 4.5 min followed by 30 cycles of denaturation at 94 °C for 45 s, annealing at 52 °C for 45 s, and extension at 72 °C for 1.5 min, followed by a final extension at 72 °C for 10 min [24]. The amplified products were sent to Shanghai Tsingke Biotechnology Co., Ltd., Shanghai, China for sequencing, and the sequences were obtained and compared with the homologous sequence information in GenBank (https://blast.ncbi.nlm.nih.gov/Blast.cgi, accessed on 1 December 2023). The homology of the sequences was analyzed by MEGA 11 and a phylogenetic tree was constructed using the Neighbor-Joining method [25].

### 2.8. Data Statistics

The mean, variance, and standard deviation of the experimental data were calculated using the software SPSS 27.0 (IBM Corp, Armonk, NY, USA), and multiple comparisons were performed using Duncan’s new multiple range test (*p* < 0.05); the data results were expressed as “mean ± standard deviation”. All images were processed using Adobe Illustrator 2022 (Adobe Systems Inc., San Francisco, CA, USA) and GraphPad Prism 8.4.3 (GraphPad Software, La Jolla, CA, USA).

## 3. Results

### 3.1. Dodder Pathogen Enrichment Culture

**Figure 1:**
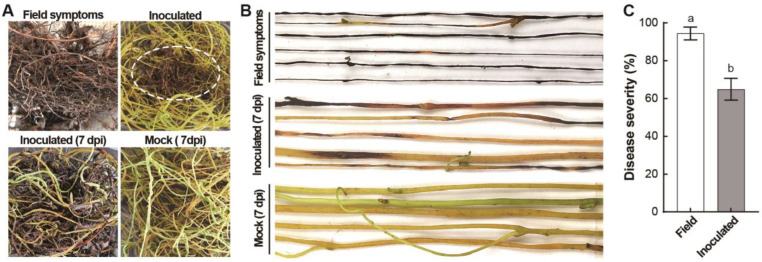
Field symptoms of diseased *C. japonica* and pathogen enrichment culture. (**A**) field symptoms of diseased *C. japonica* (white dotted circle) and pathogenicity of inoculation on *C. reflexa*. (**B**) symptoms after 7 days of pathogen enrichment culture. (**C**) differences in disease severity in the field and after inoculation. Note: letters a and b indicate significant differences between the disease indices of the tested samples.

After 7 days of inoculation with diseased tissues, the healthy dodder showed symptoms including large areas of black spots, browning, dehydration, atrophy, and a small amount of mold on the stem. With an extension in the incubation time, a large number of stems with black spots were rotted. On the other hand, the dodder sprayed with sterile water grow normally without obvious symptoms, and its stem showed a yellowish-green color with sufficient moisture content (Figure 1A,B). Meanwhile, the disease severity of the stem was 64.93% after 7 days of inoculation, suggesting that a large number of pathogens existed in the infected dodder, which could quickly infect the healthy dodder (Figure 1C).

### 3.2. Pathogens of Dodder and Their Pathogenicity

**Figure 2:**
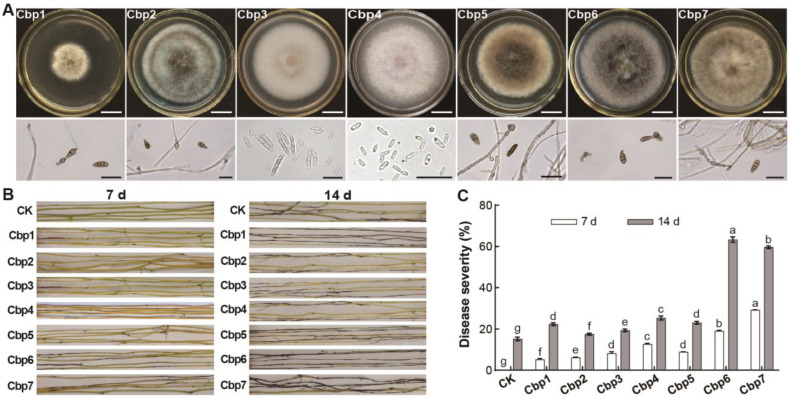
Isolation and pathogenicity determination of dodder pathogens. (**A**) morphological characteristics of pathogens (white scale bar is 1 cm, black scale bar is 20 µm). (**B**) differences in the pathogenicity of pathogens. (**C**) differences in disease severity after inoculation with pathogens. Note: letters a–g indicate significant differences between the disease indices of the tested samples.

By comparing the colony morphology, a total of 17 pure cultures were isolated from the infected samples, with 11 isolates from the naturally infected samples from the field and 6 isolates from the samples infected by enrichment inoculation. After preliminary inoculation, 7 isolates were significantly pathogenic to *C. reflexa* and numbered as Cbp1—Cbp7. Based on their morphological characteristics, it was initially determined that most of the isolated strains were *Alternaria* spp. and *Fusarium* spp. (Figure 2A). After inoculation with spore liquid spray, all 7 isolates were able to cause disease in *C. reflexa*, with dark brown lesions of varying lengths appearing on the stem, and the whole section of the stem becoming wilted and dehydrated. Colonies treated with isolates displayed the same characteristics as those obtained after re-isolation. Among them, the symptoms were more obvious after inoculation with Cbp6 and Cbp7, showing a large number of lesions appearing on the middle of the stems in addition to the black-brown lesions on the ends. In contrast, the dodder sprayed with the water control grow well and did not show obvious symptoms in the stem except for the blackening and constriction at both ends (Figure 2B). Comparison of the disease severity showed that Cbp6 and Cbp7 were the most pathogenic to *C. reflexa* with disease severity of 63.33% and 59.67% obtained based on re-isolation, respectively, which were significantly higher than the other isolates (Figure 2C). 

### 3.3. Differences in the Pathogenicity of Dodder Pathogens

**Figure 3:**
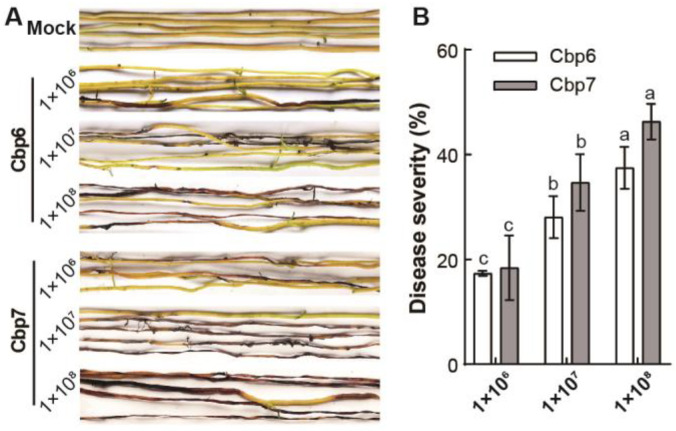
Differences in the pathogenicity of the dodder pathogens Cbp6 and Cbp7. (**A**) pathogenicity of different concentrations of spore suspensions of pathogens Cbp6 and Cbp7 on *C. reflexa*. (**B**) differences in disease severity of Cbp6 and Cbp7 on *C. reflexa.* Note: letters a–c indicate significant differences between the disease indices of the tested samples.

Higher concentrations of spore suspensions of the pathogen were found to be more pathogenic to *C. reflexa*. After 7 days of inoculation, *C. reflexa* cultured with water was healthy and strong, with yellowish-green stems and no obvious lesions. While *C. reflexa* stems sprayed with different concentrations of spore suspensions of Cbp6 and Cbp7 showed large black-brown lesions, wilting, and dehydration. Further, these symptoms were more obvious with an increase in inoculation concentration; at higher concentrations, stem segments started rotting (Figure 3A). After inoculation with a concentration of 1 × 10^8^ spores/mL of Cbp7, the disease severity was 46.30% which was slightly higher than that of Cbp6 (37.46%) (Figure 3B).

### 3.4. Physiological Characterization of Dodder Pathogens

**Figure 4:**
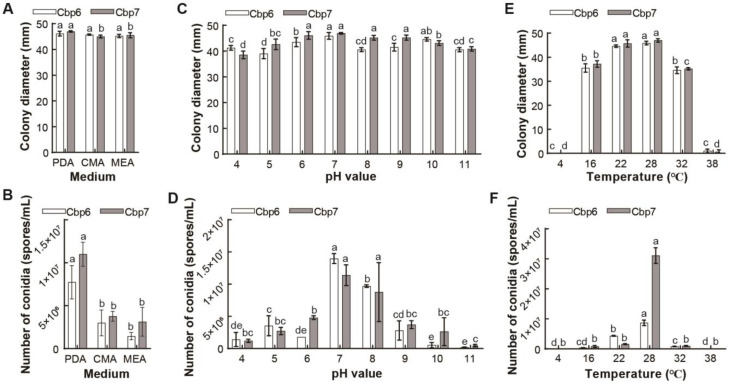
Physiological characterization of dodder pathogens. Colony diameter (**A**) and conidia production (**B**) of Cbp6 and Cbp7 on different media. Colony diameter (**C**) and conidia production (**D**) of Cbp6 and Cbp7 under different pH values. Colony diameter (**E**) and conidia production (**F**) of Cbp6 and Cbp7 under different temperature conditions. Note: letters a–e indicate significant differences between the disease indices of the tested samples.

Dodder pathogens Cbp6 and Cbp7 were able to grow on the test media, and the colony diameter was the largest on the PDA medium. The quantities of conidia from both pathogens were significantly higher on the PDA medium than on the other media. Therefore, PDA medium is optimal for the mycelium growth and the production of conidia by pathogens Cbp6 and Cbp7 (Figure 4A,B). In the pH value range of 4–11, the colonies of both pathogens grow rapidly and produced the most conidia at pH 7, while they grow slower and produced fewer conidia under acidic and alkaline conditions (Figure 4C,D). The colonies of Cbp6 and Cbp7 grow best at 22–28 °C, but most conidia were produced at 28 °C, while low or high temperatures were unfavorable for their growth and conidia production (Figure 4E,F).

### 3.5. Pathogenic Range of Dodder Pathogens

**Figure 5:**
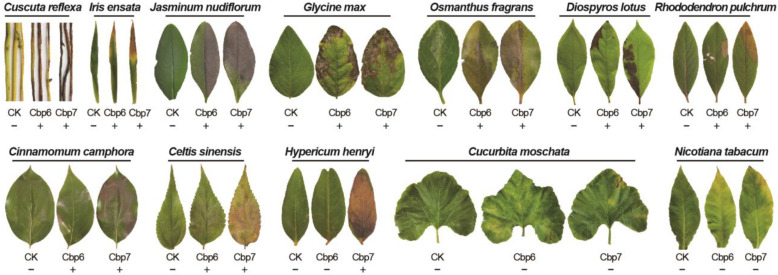
Pathogenicity of dodder pathogens on common plants. − not pathogenic; + pathogenic.

After the inoculation of leaves from 50 plant species, the pathogens from leaves showing symptoms were re-isolated, and the isolates were morphologically consistent with the inoculated pathogen colonies. It was verified that the pathogenicity spectrum of pathogen Cbp7 was wider, and it was pathogenic towards 9 plant species, namely: *I. ensata*, *J. nudiflorum*, *G. max*, *O. fragrans*, *D. lotus*, *R. pulchrum*, *C. camphora*, *C. Sinensis*, and *H. henryi,* but not *C. reflexa*. However, Cbp6 was not pathogenic to *H. henryi*, and was less pathogenic than Cbp7 to the other plant leaves. In addition to this, *C. moschata* and *N. tabacum* leaves showed slight yellowing after inoculation, whereas the other uninfected leaves were healthier and had no lesions (Figure 5).

### 3.6. Efficacy of Pathogens against Dodder in the Field

**Figure 6:**
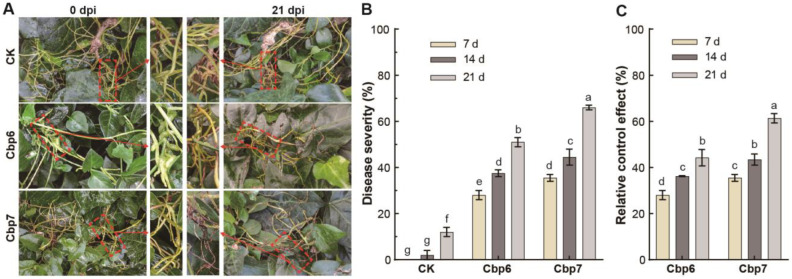
Efficacy of pathogens against dodder in the field. (**A**) pathogenicity of pathogen Cbp6 and Cbp7 on *C. reflexa* in the field. (**B**) disease severity of Cbp6 and Cbp7 on *C. reflexa*. (**C**) relative control effect of Cbp6 and Cbp7 against *C. reflexa.* Note: letters a–g indicate significant differences between the disease indices of the tested samples.

After spray inoculation, dodder in the field also showed the symptoms described before; most of the stems had a dark brown color, dried, and died after shriveling due to dehydration. Especially in the shaded and moist places of the inoculation site, the symptoms of mildew appeared (Figure 6A). After inoculation with Cbp7, the disease severity of C. reflexa reached 44.5% and 66.0% at 14 and 21 days, respectively. Compared with indoor inoculation, the outdoor environmental conditions were relatively complex, and the disease severity of outdoor inoculation was slightly lower than that of indoor, and delayed mortality in dodder was also noted (Figure 6B). The pathogen Cbp7 provided better control of C. reflexa, with a 61.3% relative control effect at 21 days, and the lesions still spread (Figure 6C).

### 3.7. Identification of Dodder Pathogens

**Figure 7:**
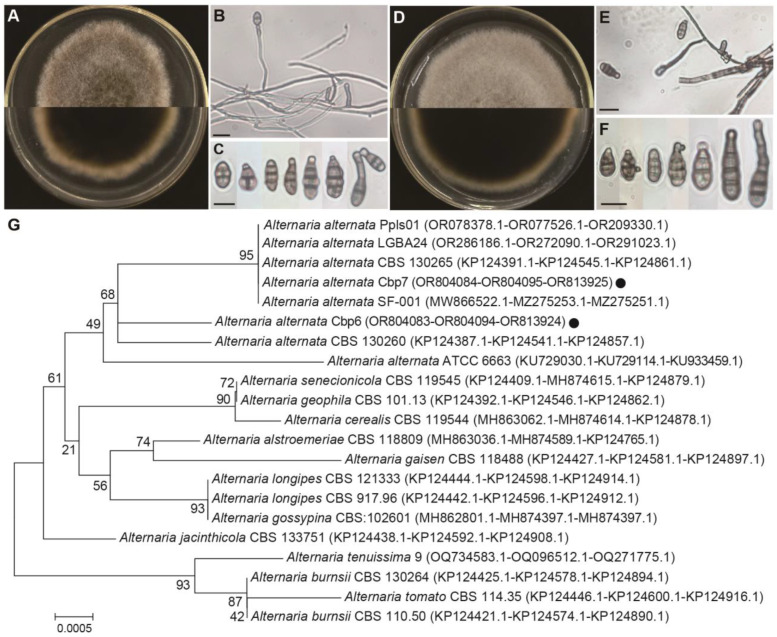
Morphological characterization and target gene sequencing identification of dodder pathogens. (**A**) Colony morphology of Cbp6. (**B**) Conidiophore of Cbp6. (**C**) Conidia of Cbp6. (**D**) Colony morphology of Cbp7. (**E**) Conidiophore of Cbp7. (**F**) Conidia of Cbp7. (**G**) Phylogenetic tree based on jointing ITS, *LSU*, and *RPB2* sequences of Cbp6 and Cbp7 (black dot indicates the dodder pathogens in this study). Note: scale bars in the images are 10 µm.

Pathogens Cbp6 and Cbp7 grow rapidly on PDA plates, with fluffy colonies and well-developed aerial mycelia. The colony colors of Cbp6 and Cbp7 were tan and gray-brown, respectively. The conidia were dark, with longitudinal and transversal septa, inverted clavate, ellipsoid, or ovate shaped, and with sizes ranging from 5–18 × 8–35 μm (average of 11.5 × 21.5 μm) (Figure 7A–F). The results of sequencing and BLAST homology comparison showed that the ITS, *LSU*, *RPB2,* and *TEF-1α* sequences of the pathogens Cbp6 and Cbp7 were most closely related to *Alternaria alternata* or *A. tenuissima*, respectively, in the GenBank database. Since there were few strains containing *TEF-1α* sequences, a phylogenetic tree was constructed by joining the ITS, *LSU*, and *RPB2* sequences, and the results of the phylogenetic tree also confirmed that Cbp6 and Cbp7 were most closely related to *A. alternata* (Figure 7G). A phylogenetic tree based on the *TEF-1α* gene is shown in the Supplementary Figure S1. After combining the molecular and morphological characteristics, Cbp6 and Cbp7 were identified as *A. alternata*. All of the above sequencing results have been submitted to the NCBI GenBank database, and the sequence accession numbers are listed in Figure 7G.

## 4. Discussion

Dodder has intense growth competition with crops and horticultural plants and limits agricultural production resulting in serious economic losses [8]. Chemical herbicides such as glyphosate, dicamba, and 2,4-dichlorophenoxyacetic acid (2,4-D) cannot be used to control parasitic plants. This is because dodder forms a symbiotic relationship with the host plant and can readily transfer herbicide resistance or acquire systemic signaling substances from the host plant in response to insect feeding, pathogenic fungal infestation, and other various kinds of stress [16,26,27,28]. Biological herbicides can efficiently control herbicide-resistant weeds while protecting ecosystem functioning and biodiversity in line with the concept of sustainable agriculture; therefore, they are ideal for the long-term control of dodder and other agricultural weeds [29].

Common plant pathogenic fungi such as *Colletotrichum* spp., *Phoma* spp., *Alternaria* spp., *Fusarium* spp., *Curvularia* spp., *Cercospora* spp., *Trichoderma* spp., and *Puccinia* spp. have been used as bioherbicides to control parasitic plants and weeds with good control effect [30,31,32,33,34]. In this study, a total of 7 dodder strains related to diseases were isolated based on colony morphology and microstructure. *Alternaria* spp. was most frequently isolated followed by *Fusarium* spp., known as the most common and destructive plant pathogens [35,36]. When comparing pathogenicity, Cbp6 and Cbp7 were the most pathogenic, demonstrating good control of dodder in both in vitro and in vivo inoculations. It has been shown that pathogenic fungi such as *F. semitectum*, *F. solani*, *A. tenuissima*, and *Pseudopestalotiopsis theae* are pathogenic to *C. reflexa* [37]. The above results demonstrate that *Alternaria* spp. is a dominant pathogenic fungus of dodder and has the potential to be used as a bioherbicide.

Additionally, plant pathogens may adversely affect crop production and forest health; therefore, before plant pathogens are used as bioherbicides they should be subjected to comprehensive host-specific testing to assess the effects they produce in target weeds and any risks they pose to non-target plants [18]. *Alternaria* spp. are common plant pathogens that cause leaf spot or wilt in a large number of horticultural plants and crops, endangering agricultural production [38,39]. However, it was confirmed that Cbp6 and Cbp7 were not significantly pathogenic to most of the plants, except for *I. ensata*, *J. nudiflorum*, and *G. max*. Meanwhile, *C. reflexa* parasitizing on *H. nepalensis* var. *sinensis* and *V. major* was controlled with highly pathogenic isolates. The pathogens showed efficient control of *C. reflexa* with wilting and death, while no disease was seen in the host plants, confirming that Cbp6 and Cbp7 are highly specific for *C. reflexa,* and have a high level of biosafety for horticultural plants and a low impact on the ecosystem.

According to statistics, there are more than 200 species of dodder in the world, and 14 species are growing in China. Among them, the most serious and common ones are *C. reflexa*, *C. chinensis*, *C. australis,* and *C. japonica* [1,40]. The favorable climatic conditions and rich vegetation in Yunnan Province, China provide ideal conditions for dodder to parasitize other plants. *C. reflexa* infects 181 plants of 66 families and 161 genera in this area, and therefore it is one of the most abundant species [40]. Dodder grows vigorously under suitable environmental conditions in the field and generally dies slowly after the host plant has completely withered and died because its weak photosynthesis is insufficient to sustain the physiological needs [3].

## 5. Conclusions

In conclusion, the pathogens isolated from the dead dodder species, *C. reflexa* and *C. japonica* effectively, while they were not pathogenic to the weeds’ host plants, and can be regarded as biologically safe. Therefore, they have great potential for the biocontrol of dodders. Finally, our results provide a theoretical and experimental foundation for the prevention and control of dodders through the application of naturally occurring native pathogens.

## Data Availability

The original contributions presented in the study are included in the article/Supplementary Material, further inquiries can be directed to the corresponding authors.

## Supplementary Materials

The following supporting information can be downloaded at: https://www.mdpi.com/article/10.3390/jof10070494/s1, Figure S1: Phylogenetic tree based on *TEF-1α* sequences of Cbp6 and Cbp7.

## References

[1] Shen G., Liu N., Zhang J., Xu Y., Baldwin I.T., Wu J. (2020). *Cuscuta australis* (dodder) parasite eavesdrops on the host plants’ FT signals to flower. Proc. Natl. Acad. Sci. USA.

[2] Sun G., Xu Y., Liu H., Sun T., Zhang J., Hettenhausen C., Shen G., Qi J., Qin Y., Li J. (2018). Large-scale gene losses underlie the genome evolution of parasitic plant *Cuscuta australis*. Nat. Commun..

[3] Hartenstein M., Albert M., Krause K., Lunn J. (2023). The plant vampire diaries: A historic perspective on *Cuscuta* research. J. Exp. Bot..

[4] Jhu M.-Y., Sinha N.R. (2022). *Cuscuta* species: Model organisms for haustorium development in stem holoparasitic plants. Front. Plant Sci..

[5] Kim G., Westwood J.H. (2015). Macromolecule exchange in *Cuscuta*–host plant interactions. Curr. Opin. Plant Biol..

[6] Shahid S., Kim G., Johnson N.R., Wafula E., Wang F., Coruh C., Bernal-Galeano V., Phifer T., dePamphilis C.W., Westwood J.H. (2018). MicroRNAs from the parasitic plant *Cuscuta campestris* target host messenger RNAs. Nature.

[7] Wu Y., Luo D., Fang L., Zhou Q., Liu W., Liu Z. (2022). Bidirectional lncRNA transfer between *Cuscuta* parasites and their host plant. Int. J. Mol. Sci..

[8] Masanga J., Mwangi B.N., Kibet W., Sagero P., Wamalwa M., Oduor R., Ngugi M., Alakonya A., Ojola P., Bellis E.S. (2021). Physiological and ecological warnings that dodders pose an exigent threat to farmlands in Eastern Africa. Plant Physiol..

[9] Shen H., Hong L., Ye W., Cao H., Wang Z. (2007). The influence of the holoparasitic plant *Cuscuta campestris* on the growth and photosynthesis of its host *Mikania micrantha*. J. Exp. Bot..

[10] Wu A.P., Zhong W., Yuan J.R., Qi L.Y., Chen F.L., Liang Y.S., He F.F., Wang Y.H. (2019). The factors affecting a native obligate parasite, *Cuscuta australis*, in selecting an exotic weed, *Humulus scandens*, as its host. Sci. Rep..

[11] Ahmad A., Tandon S., Xuan T.D., Nooreen Z. (2017). A review on phytoconstituents and biological activities of *Cuscuta* species. Biomed. Pharmacother..

[12] Ibrahim M., Rehman K., Hussain I., Farooq T., Ali B., Majeed I., Akash M.S.H. (2017). Ethnopharmacological investigations of phytochemical constituents isolated from the genus *Cuscuta*. Crit. Rev. Eukaryot. Gene Expr..

[13] Shimizu K., Aoki K. (2019). Development of parasitic organs of a stem holoparasitic plant in genus *Cuscuta*. Front. Plant Sci..

[14] Fürst U., Hegenauer V., Kaiser B., Körner M., Welz M., Albert M. (2016). Parasitic *Cuscuta* factor(s) and the detection by tomato initiates plant defense. Commun. Integr. Biol..

[15] Johnsen H.R., Striberny B., Olsen S., Vidal-Melgosa S., Fangel J.U., Willats W.G.T., Rose J.K.C., Krause K. (2015). Cell wall composition profiling of parasitic giant dodder (*Cuscuta reflexa*) and its hosts: A priori differences and induced changes. New Phytol..

[16] Jiang L., Qu F., Li Z., Doohan D. (2013). Inter-species protein trafficking endows dodder (*Cuscuta pentagona*) with a host-specific herbicide-tolerant trait. New Phytol..

[17] Moreno-Robles A., Cala Peralta A., Zorrilla J.G., Soriano G., Masi M., Vilariño-Rodríguez S., Cimmino A., Fernández-Aparicio M. (2022). Identification of structural features of hydrocinnamic acid related to its allelopathic activity against the parasitic weed *Cuscuta campestris*. Plants.

[18] Chaudhury F.A., Khan I.H., Javaid A. (2023). Effect of quinoa biomass and biocontrol fungi on expression of IPER gene in mung bean in *Macrophomina phaseolina* contaminated soil. Adv. Life Sci..

[19] Bailey K.L. (2014). The bioherbicide approach to weed control using plant pathogens. Integrated Pest Management.

[20] Liu Y., He P., He P., Munir S., Ahmed A., Wu Y., Yang Y., Lu J., Wang J., Yang J. (2022). Potential biocontrol efficiency of *Trichoderma* species against oomycete pathogens. Front. Microbiol..

[21] White T.J., Bruns T., Lee S., Taylor J. (1990). Amplification and direct sequencing of fungal ribosomal RNA genes for phylogenetics. PCRProtoc. Guide Methods Appl..

[22] Stielow J.B., Lévesque C.A., Seifert K.A., Meyer W., Irinyi L., Smits D., Renfurm R., Verkley G.J.M., Groenewald M., Chaduli D. (2015). One fungus, which genes? Development and assessment of universal primers for potential secondary fungal DNA barcodes. Persoonia—Mol. Phylogeny Evol. Fungi.

[23] Matheny P.B. (2005). Improving phylogenetic inference of mushrooms with RPB1 and RPB2 nucleotide sequences (*Inocybe*; Agaricales). Mol. Phylogenetics Evol..

[24] Liu Y., Gui T., Ahmed A., Munir S., He P., He P., Wu Y., Tang P., Luo Q., He Y. (2024). *Pyricularia pennisetigena* as leaf blast disease-causing pathogen in king grass (*Pennisetum sinese*) and its assessment of the pathogenic risk. J. Plant Pathol..

[25] Liu Y., Ahmed A., Munir S., He P., He P., Wu Y., Tang P., Wang Z., Kong B., He Y. (2023). First report of *Aloe* root and stem rot caused by *Phytophthora palmivora* in Yunnan Province, China. Plant Dis..

[26] Hartung J.S., Paul C., Achor D., Brlansky R.H. (2010). Colonization of dodder, *Cuscuta indecora*, by ‘*Candidatus* Liberibacter asiaticus’ and ‘*Ca*. L. americanus’. Phytopathology.

[27] Li J., Hettenhausen C., Sun G., Zhuang H., Li J.-H., Wu J. (2015). The parasitic plant *Cuscuta australis* is highly insensitive to abscisic acid-induced suppression of hypocotyl elongation and seed germination. PLoS ONE.

[28] Qin Y., Zhang J., Hettenhausen C., Liu H., Li S., Shen G., Cao G., Wu J. (2019). The host jasmonic acid pathway regulates the transcriptomic changes of dodder and host plant under the scenario of caterpillar feeding on dodder. BMC Plant Biol..

[29] Triolet M., Edel-Hermann V., Gautheron N., Mondy S., Reibel C., André O., Guillemin J.-P., Steinberg C., Druzhinina I.S. (2022). Weeds harbor an impressive diversity of fungi, which offers possibilities for biocontrol. Appl. Environ. Microbiol..

[30] Den Breeyen A., Lange C., Fowler S.V. (2022). Plant pathogens as introduced weed biological control agents: Could antagonistic fungi be important factors determining agent success or failure?. Front. Fungal Biol..

[31] Radhakrishnan R., Alqarawi A.A., Abd Allah E.F. (2018). Bioherbicides: Current knowledge on weed control mechanism. Ecotoxicol. Environ. Saf..

[32] Rai M., Zimowska B., Shinde S., Tres M.V. (2021). Bioherbicidal potential of different species of *Phoma*: Opportunities and challenges. Appl. Microbiol. Biotechnol..

[33] Shi B., Osunkoya O.O., Soni A., Campbell S., Dhileepan K. (2022). Growth of the invasive Navua sedge (*Cyperus aromaticus*) under competitive interaction with pasture species and simulated grazing conditions: Implication for management. Ecol. Res..

[34] Zhu H., Ma Y., Guo Q., Xu B. (2020). Biological weed control using *Trichoderma polysporum* strain HZ-31. Crop Prot..

[35] Dean R., Van Kan J.A.L., Pretorius Z.A., Hammond-Kosack K.E., Di Pietro A., Spanu P.D., Rudd J.J., Dickman M., Kahmann R., Ellis J. (2012). The Top 10 fungal pathogens in molecular plant pathology. Mol. Plant Pathol..

[36] Pinto V.E.F., Patriarca A. (2017). Alternaria species and their associated mycotoxins. Mycotoxigenic Fungi.

[37] Gou F.G., Li Y.H., Deng F.Z. (1998). Screening biological control fungi for dodders on woody hosts. Chin. J. Biol. Control.

[38] DeMers M. (2022). *Alternaria alternata* as endophyte and pathogen. Microbiology.

[39] Ivanović Ž., Blagojević J., Jovanović G., Ivanović B., Žeželj D. (2022). New insight in the occurrence of early blight disease on potato reveals high distribution of *Alternaria solani* and *Alternaria protenta* in Serbia. Front. Microbiol..

[40] Zhang F., Yue Y., Shen S., Xu G., Guo J., Zhang Y. (2017). The control effect of *Cuscuta* on *Mikania micrantha* and their ecological index in Yunnan. Ecol. Environ. Sci..

