# Supplementary figures and images for: Alternaria alternata Pathogen from Cuscuta japonica Could Serve as a Potential Bioherbicide
Source: J Fungi (Basel). 2024 Jul 17;10(7):494. doi: 10.3390/jof10070494 (PMC11277819; doi:10.3390/jof10070494)

Supplementary figure 1

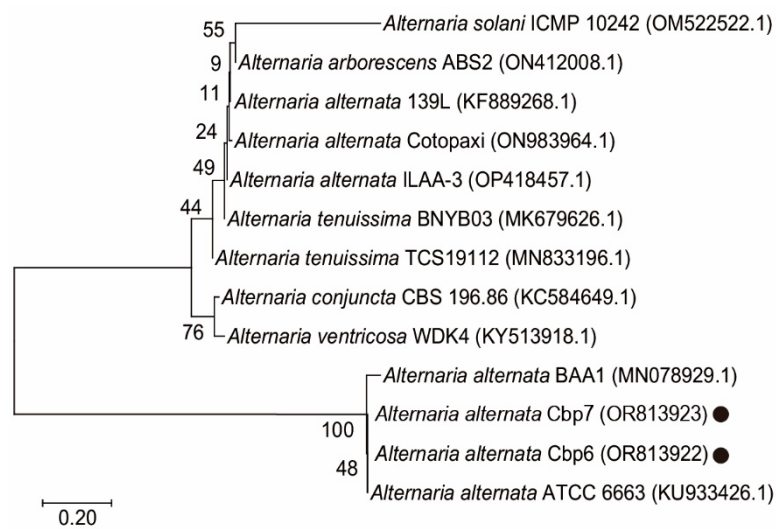

Figure S1. Phylogenetic tree based on *TEF-1α* sequences of Cbp6 and Cbp7.

Supplement: Supplementary file 1 [file jof-10-00494-s001.zip › jof-3067698-supplementary.pdf]
